# A Soil-Plate Based Pipeline for Assessing Cereal Root Growth in Response to Polyethylene Glycol (PEG)-Induced Water Deficit Stress

**DOI:** 10.3389/fpls.2017.01272

**Published:** 2017-07-19

**Authors:** Sven K. Nelson, Melvin J. Oliver

**Affiliations:** United States Department of Agriculture–Agricultural Research Services and Interdisciplinary Plant Group, University of Missouri, Columbia MO, United States

**Keywords:** water deficit, wheat, rhizobox, abiotic stress, root, soil, PEG

## Abstract

Drought is a serious problem that causes losses in crop-yield every year, but the mechanisms underlying how roots respond to water deficit are difficult to study under controlled conditions. Methods for assaying root elongation and architecture, especially for seedlings, are commonly achieved on artificial media, such as agar, moistened filter paper, or in hydroponic systems. However, it has been demonstrated that measuring root characteristics under such conditions does not accurately mimic what is observed when plants are grown in soil. Morphological changes in root behavior occur because of differences in solute diffusion, mechanical impedance, exposure to light (in some designs), and gas exchange of roots grown under these conditions. To address such deficiencies, we developed a quantitative method for assaying seedling root lengths and germination in soil using a plate-based approach with wheat as a model crop. We also further developed the method to include defined water deficits stress levels using the osmotic properties of polyethylene glycol (PEG). Seeds were sown into soil-filled vertical plates and grown in the dark. Root length measurements were collected using digital photography through the transparent lid under green lighting to avoid effects of white light exposure on growth. Photographs were analyzed using the cross-platform ImageJ plugin, SmartRoot, which can detect root edges and partially automate root detection for extraction of lengths. This allowed for quick measurements and straightforward and accurate assessments of non-linear roots. Other measurements, such as root width or angle, can also be collected by this method. An R function was developed to collect exported root length data, process and reformat the data, and output plots depicting root/shoot growth dynamics. For water deficit experiments, seedlings were transplanted side-by-side into well-watered plates and plates containing PEG solutions to simulate precise water deficits.

## Introduction

Although drought is a widespread problem that causes losses in yield for crops each year, advances in drought tolerance have been slow to emerge (reviewed in Lawlor, 2013). Since water uptake occurs in the roots, root traits present promising targets for drought adaptation. Understanding the mechanisms of root responses to water-deficit stress is key to the development of targeted strategie for improving drought tolerance in important crops, e.g., wheat. However, many research studies investigating crop responses to drought focus only on above-ground effects due, in part, to the difficulties associated with assaying parts of the plant that are below ground (Maeght et al., 2013). For roots grown in soil, assaying such traits involves “shovelomics” — exhuming and cleaning the roots — which is labor intensive, time-consuming, destructive, and prohibits consecutive time-course studies of the same roots. If roots are grown in media for real-time observations, care must be taken to keep roots dark as activation of red and blue light receptors can inhibit root growth when roots are exposed to white light (Kutschera and Briggs, 2012; Yokawa et al., 2014; Mo et al., 2015; Lee et al., 2016). Experiments performed on filter paper, agar, or in hydroponics all present artifacts relating to the use of a liquid-based media that confound the interpretation of the data (Rich and Watt, 2013; Robbins and Dinneny, 2015). Alternatively, experiments using soil are often conducted without precise quantification of the level of water-deficit stress imposed, which precludes accurate interpretation of the data, as low and high water deficit stress can have the opposite effects on root growth (Morgan, 1984; Sharp and Davies, 1989). Controlled experiments with known water stress conditions, ideally by measuring the substrate water potential (ψ_w_), are necessary to generate meaningful comparisons between studies (Lawlor, 2013; Robbins and Dinneny, 2015).

Most of the seminal works in understanding the regulation of seedling root growth under controlled water stress conditions have been conducted in the C4 monocot *Zea mays* (maize), primarily using a protocol where Plexiglass boxes are filled with vermiculite under carefully controlled water deficit, light, and humidity conditions (Silk et al., 1986; Sharp et al., 1988, 1990, 2004; Saab et al., 1990; Sacks et al., 1997; Yamaguchi and Sharp, 2010). When employed under controlled conditions, these growth boxes (sometimes called rhizoboxes) containing soil or soil-like growth substrates provide a viable hybrid between shovelomics and the use of liquid-based media. Seedlings grown with roots against a transparent wall allow observations without excavation. In such experiments, maize seedlings are grown under well-watered (WW) conditions and then transferred to boxes containing vermiculite either still WW or at specific and defined water-deficit stresses (WS) as quantified by psychrometric measurements of the vermiculite ψ_w_ (Seeve et al., 2016). Plant growth occurs in a dark room at humidity conditions near saturation to minimize transpiration by the seedlings, which could affect soil water content. Handling of seedlings and experimental observations are made under a green-safe light to prevent any light effects on root growth. This methodology has facilitated comparisons of root growth under highly controlled water stress conditions in the maize seedling primary root.

**FIGURE 1:**
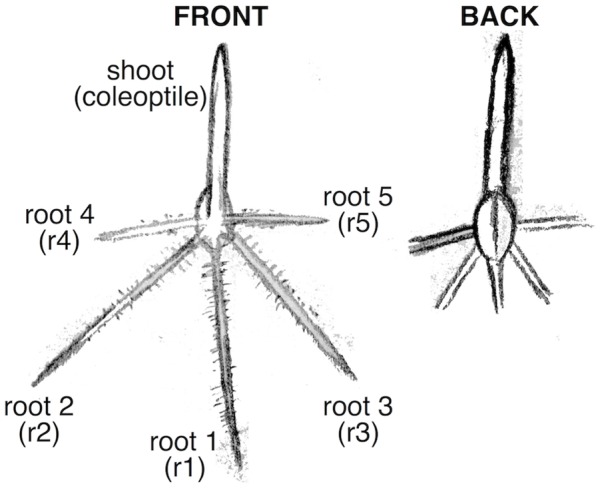
Diagram of wheat seedling root organization. The coleoptile (shoot) and roots 1–3 emerge in close succession. Roots 4 and 5 emerge later. Some wheat varieties have a 6th root.

This foundational research in maize paved the way for research to understand water stress root responses in other crop species (Sharp et al., 2004; Yamaguchi and Sharp, 2010; Voothuluru et al., 2013; Nagel et al., 2015). Unlike maize, the remainder of the five major cereal crops, wheat, rice, barley, and oats, are all C3 monocots and are more closely related to each other than they are to maize (Kellogg, 1998). Furthermore, whilst maize and rice have root systems characterized by a single primary root, which is the only seminal root emerging from below the scutellar node in the seed, wheat, barley, and oats all have multiple sub-scutellar seminal roots (also called primary roots) (Avery, 1930; Rich and Watt, 2013). This means that findings from studies of the maize primary root may not be directly transferable to other cereal plant systems, particularly those where multiple primary roots are present. To understand how the roots of a particular cereal respond to water stress, it is necessary to conduct controlled experiments in that specific cereal crop. This may present novel challenges, for instance when assaying wheat seedlings by a similar method one is faced with the issue that wheat seedlings are smaller and more narrow than in maize and may not be readily visible in a rhizobox. In addition, wheat seedlings can have 5–6 primary roots that emerge at varying angles from the vertical plane (meaning fewer seedlings can fit into a rhizobox and more time is required for root measurements), the first three of which emerge almost simultaneously (Manske and Vlek, 2002; **Figure 1**). The roots are clearly distinguishable by their location of emergence, but are difficult to study independently using the current methods available. Thus, the root characteristics of wheat demand the development of new or adapted methodologies to allow quick and accurate measurements of root traits under water-deficit treatments.

Many existing methods for managing substrate water potential exposure of roots have employed polyethylene glycol (PEG) to simulate a specific water deficit exposure (van der Weele et al., 2000; Ji et al., 2014; Opitz et al., 2014; Seeve et al., 2016; Frolov et al., 2017). PEG is almost exclusively used in liquid-based systems such as wet filter paper, agar, or hydroponics. In these systems, a water deficit cannot be simulated by withholding water and so an osmoticum such as PEG is used to induce a low osmotic potential in the media thus causing water loss from the exposed root. A major confounding factor in such protocols is that, unlike in dry soil, the root system is sensing water deficit but is still physically surrounded by water which can lead to an artifactual response (Verslues et al., 1998; Ahn et al., 2004). Soil is not a solution, it is a complex matrix of particles, liquid, and air pockets (Robbins and Dinneny, 2015). Roots develop and respond differently in soil than in liquid media due to the difference in solute diffusion, mechanical impedance, and the gas exchange properties of liquid versus soil (Gahoonia and Nielsen, 1997; Genc et al., 2007; Chapman et al., 2011; Rich and Watt, 2013; Robbins and Dinneny, 2015; Lin et al., 2016). The use of PEG to simulate water deficits in soil has generally been avoided as PEG concentration and accessibility changes as the soil dries, but the use of PEG in a solid-matrix such as soil is viable when used in a non-transpiring system where the soil saturation levels remain constant. Herein we describe a plate methodology and an associated data analysis pipeline developed to assay cereal seedling root growth under controlled water deficit conditions induced using PEG in soil. The methodology was primarily developed using wheat as the cereal model.

The soil-plate method was developed to allow root growth measurements of wheat seedlings over a 3-day time-course. The protocol was designed to ensure that a precise and specific water-deficit stress is imposed by measurement of soil ψ_w_, and under controlled conditions. By using soil as the substrate and PEG to induce precise WS, the method avoids artifacts resulting from the use of liquid-media. Furthermore, the use of PEG in this fashion permits the assessment of the effects of osmotic stress independent of other factors that might otherwise vary during natural soil drying. Seedlings were grown vertically in clear-walled square plates to allow observations without exhuming. Plates were not exposed to light after the initial plating and observations made under green light to prevent effects of white light since roots contain red and blue light receptors (Yokawa et al., 2014). Green light has been used as a root-safe light source in similar studies, and although it is likely not entirely without effect on root growth, any green light-associated responses have been reported as subtle and difficult to quantify, while red and blue light-associated responses were clearly quantifiable (Tripathy and Brown, 1995; Folta and Maruhnich, 2007). For multiple replicates, each with multiple roots, manual root measurements are not efficient and do not offer an ideal method to capture root lengths at static time-points. In this protocol, seedlings were digitally photographed at each time-point and root lengths computationally determined using the SmartRoot image analysis software (Lobet et al., 2011). By extracting root lengths from images, precise measurements could be obtained even from growing non-linear roots, such as those that start horizontally and then curve downward. The R package measuRoots was developed to streamline data analysis, and includes a graphical interface to automate all post processing and analysis steps from the data output of SmartRoot. This non-invasive method to capture root length data also facilitated efficient collection of root tissue at the conclusion of the experiment for hormone or transcriptomic analyses.

## Materials and Equipment

### Preparations

**FIGURE 2:**
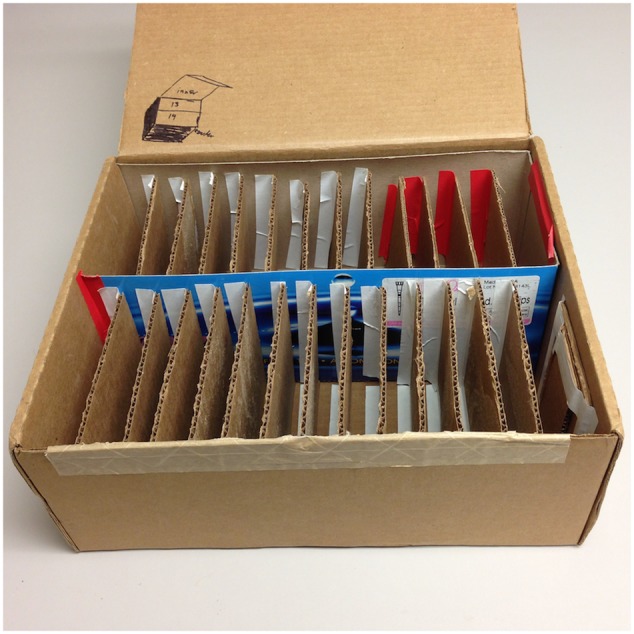
Vertical storage of plates. An example of a box with slots for vertical storage of plates in the incubator is shown.

1. **Soil:** To prevent obstruction of roots by large particles, it may be desirable to select a soil free from large organic debris or perlite, however, small particles will not greatly inhibit the ability to capture root measurements. Under our conditions, ProMix BX (Premier Tech Horticulture) potting soil mix was used, which contains perlite. Soil for experimental use should be stored in a large ziplock bag or Tupperware container in a location with stable temperature and humidity. Storage conditions should be modified if a “living” soil is used which contains soil microorganisms. Soil should be equilibrated under storage conditions for at least 1 week before use.
2. **Box with slots for vertical plates:** To store a large number of square plates in an incubator in a vertical orientation, it is necessary to construct a stand. This can be constructed from a cardboard box as shown in **Figure 2**.

3. **Camera:** Photographs were taken using an iPhone 5 with the standard camera app with the square photo setting with the grid option enabled. Any camera with sufficient resolution is suitable, however, cell phone cameras are convenient for easy data transfer and possess good autofocus and low-light correction. The flash capability should be disabled and any screen or other light producing area of the camera should be wrapped in green film to prevent exposure of roots to non-green light.
4. **Green lighting:** Green lights can either be purchased that utilize green light LEDs sold as plant photoperiod-safe green lights such as Apollo Horticulture item # AH LED-GRN-9, or can be produced by covering white lights with green light filters. In the later case, wavelength of the light passing through the filter can be determined using an inexpensive EISCO Quantitative Spectroscope (item # PH101QA) which has an accuracy of about ± 5 nm. Calibration and plotting of light spectral data can be performed using the Public Lab Spectral Workshop online software^1^. Filtered light in this experiment was confirmed to be primarily green when compared to the unfiltered light source (Supplementary Figure 1).

### Equipment

Square polystyrene plates (120 mm × 120 mm × 17 mm; Greiner bio-one item # 688102)
WP4C dewpoint hygrometer (Decagon) or similar instrument
Dark room (with green lights)
Aspirator (for seed sterilization)
Incubator (laboratory incubator capable of maintaining 28°C or desired growth temperature)
Parafilm
Aluminum foil
Lab tape
16 mm white plastic strips for internal rulers

### Solutions

1. **Sterilization solution:** 10% Bleach (6.0% sodium hypochlorite solution) and 0.01% SDS in water.
2. **500 mM MES, pH 5.5:** To prepare a 500 mL stock solution, dissolve 48.81 g of 2-(*N*-morpholino)ethanesulfonic acid (MES) into approximately 400 mL of ddH_2_O. Mix and adjust the pH to 5.5, and bring to 500 ml with water, re-mix and re-adjust pH. Filter sterilize and store at 4°C.
3. **WW soil stock solution (5 mM MES):** Prepare 1 L of 5 mM MES solution by adding 10 mL of 500 mM MES to 990 mL of ddH_2_O.
4. **WS soil stock solution (20% PEG in 5 mM MES):** Prepare 1 L of 20% PEG WS solution by weighing out 200 mg of PEG-8000 and adding to 790 mL ddH_2_O and 10 mL 500 mM MES. Mix well as PEG may take a little while to dissolve. It is not necessary to re-test the pH at this step. Different concentrations of PEG may be used for greater or lesser osmotic stress treatments.

## Protocol Overview

(I) Sterilize seeds for plating.
(II) Prepare WW soil for three plates of 45 seeds each.
(III) Plate seeds and measure the water potential of the soil.
(IV) Wrap sealed plates in foil to keep dark and place vertically in incubator for 30 h at 28°C.
(V) Prepare 3 WW and 3 WS soil plates for transplanting and measure the water potentials.
(VI) At 30 h, transplant eight seedlings per plate to WW and WS plates under green light (dark) conditions.
(VII) Photograph seedlings under green light (dark).
(VIII) Wrap sealed plates in foil to keep dark and place vertically in incubator for 24 h at 28°C.
(IX) At 24 h after transplanting, photograph seedlings under green light (dark).
(X) Wrap in foil and replace in incubator (vertically) for another 24 h at 28°C.
(XI) At 48 h after transplanting, photograph seedlings individually on soil plates under green light. Samples can be collected here for biochemical or molecular analyses.
(XII) Use imageJ and the SmartRoot plugin to perform “root calling” to produce CSV data files.
(XIII) Use functions from the custom-built **measuRoots** R package to process and plot the data.

## Stepwise Procedures

This soil-plate method facilitates straightforward, quantitative, and accurate assessment of root growth while maintaining the use of soil. An experiment comparing WS and WW seedling root growth would consist of three plates containing eight seedlings per plate for a total of 24 replicates for each treatment. To ensure that seedlings are at a comparable developmental stage and that un-germinated seeds do not influence results, seeds are first germinated on WW soil and seedlings that have reach an equivalent stage in development after 30 h of growth are chosen and transferred to WW and WS treatments. For wheat, at the time of transfer the coleoptile (shoot) and roots 1, 2, and 3 should have emerged with root 1 at approximately 10 mm of length. Root 4 and 5 must not be present at the time of transfer. Starting with 3 plates with 45 seeds each for germination will ensure that enough seedlings are at the appropriate developmental stage at the time of transplant. Since soil-plates are quick and easy to prepare, and collecting measurements is as simple as taking a digital photograph, the difference between measuring a single soil-plate or a large number of soil-plates is relatively low allowing experiments to be scaled up as desired.

### Seed Surface Sterilization

(1) Place seeds in 15 mL conical tubes, with a maximum of 45 seeds per tube.
(2) Add sterilization solution 5–10 mL above the level of the dry seeds. Tightly screw lid.
(3) Shake every 5 min for 15 min (or more often).
(4) Use vacuum aspirator to remove the solution.
(5) Add approximately 10 mL of sterile water to the tube.
**NOTE:** It may be useful to record the time here as the start of imbibition.
(6) Shake every few minutes for 7 min.
(7) Aspirate off the water and repeat from step 5–7 about 3–4 times until there is no bleach odor detectable from the tube.
(8) Recap the tube and prepare soil for plating.

### Preparation of Soil with Known Water Potential

Seeds for both the WW and WS treatments will be plated on WW plates for germination and then transplanted to WW or WS plates after 30 h to ensure all seedlings used for comparisons are germinated and at a similar developmental stage at the start of treatment.

(1) Before measuring out soil to prepare plates, always mix the supply thoroughly by shaking the storage container/bag. This ensures that the soil sampled will be generally representative of all of the soil in the supply, decreasing the likelihood of plate-to-plate differences.
(2) Measure out the soil needed for three WW soil-plates for germination. As a rule of thumb, 30 grams of soil is needed per plate to ensure enough soil to fill the plate and have some remaining for water potential measurement.
(3) Place the soil in a large zip-lock bag pre-labeled with the soil condition (WW) and date.
(4) Add 3 × the soil mass as liquid volume of WW soil solution (5 mM MES). For three plates, that equals 270 mL of solution. This ratio may need to be adjusted based on soil composition.
(5) Seal the bag and mix well by shaking and kneading thoroughly. The soil is now ready to use.
(6) Measure water potential by taking a small sample from the bag and adding to one of the metal sample cups from the WP4C dewpoint hygrometer.
**NOTE:** Soil water potential measurements should be taken in “precise mode” and using metal sample cups for accurate measurements under the conditions used in this experiment. The WP4C dewpoint hygrometer should be calibrated at the beginning of each experiment using the 0.5 mol/kg KCL calibration solution provided by the manufacturer.
(7) Insert the cup into the instrument and begin a water potential measurement. Take three readings of this sample to better account for the proportion of variance attributed to error. Begin plating while the dewpoint hygrometer is taking measurements, as each measurement may take about 10–15 min.

### Plating Seeds onto Soil-Plates

**FIGURE 3:**
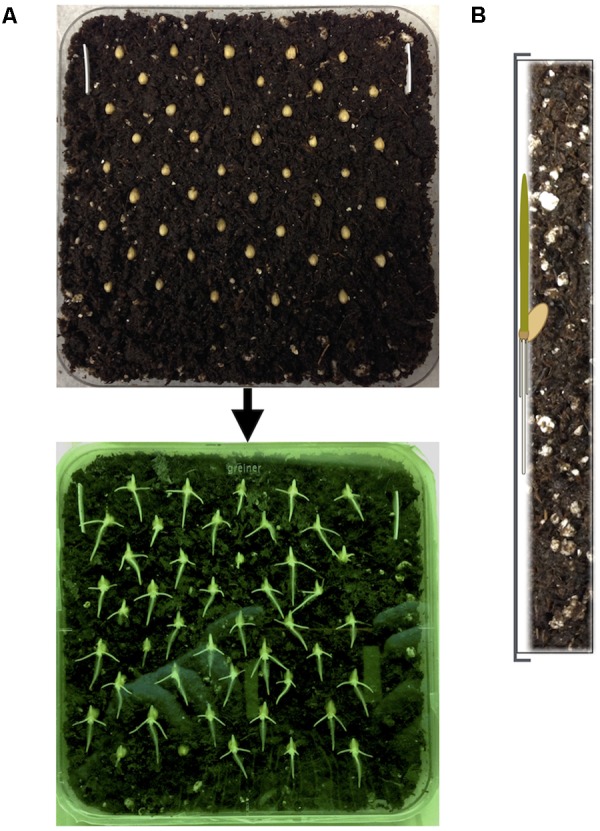
Arrangement of seedlings in soil-plates. **(A)** Seedlings are sterilized and plated into soil-plates in staggered rows of five. A 16 mm plastic strip is inserted into the soil to serve as a scale during analysis of digital photographs. Seedlings are germinated vertically and transplanted at 30 h by selecting only seedlings at the same developmental stage. **(B)** Representation of the plate side view containing a single seedling. Seed angle is important for wheat during planting so that roots and shoot emerge at the same plane as the surface of the soil and the transparent plate lid.

(1) Label the back of each plate and add the prepared soil with gloved hands until each plate is filled to the top. Soil should be flush with the top of the plate and packed enough to leave no large airspaces in the soil, but not so much as to create pressure against the lid when the plate is closed.
**NOTE:** If you used a potting mix that contains larger organic particles or perlite, remove large particles manually at this step or by passing the soil through a mesh prior to soil preparation.
(2) Insert two to four 16 mm (or other known length) white plastic strips into the soil at either end of the plate (**Figure 3A**). The top of these strips should be flush with the surface of the soil. These strips will provide a length reference in images for the measurement of root growth.
(3) Insert surface sterilized seeds into the soil using forceps in the arrangement indicated in **Figure 3A**. The diagram in Supplementary Figure 2 is provided to serve as a to-scale guide for seed placement.
(a) Seeds should be inserted in nine rows of five seeds per row. Each row should be staggered relative to the row above and below to minimize root-shoot overlap. Prepare 3 plates of 45 seeds each to allow enough to select 48 seedlings at a uniform developmental state after 30 h for transplant into WW and WS soil-plates.
(b) For wheat, each grain should be inserted crease-inward at about a 30° angle from the surface of soil, such that the embryo end is exposed and will point downward when the plate is vertical (**Figure 3B**). This angle is important for wheat grains to allow the seminal roots to emerge parallel with the soil surface and also to prevent the shoot from getting compressed between the grain and the plastic.
(4) Lid plates and hold vertically to ensure seeds are held in place.
(6) Seal each plate with 3–6 strips of parafilm to keep moisture in.
(6) Wrap each plate in aluminum foil to keep out light, keeping track of the orientation. Wrapping a single piece of lab tape around the edge over the parafilm before wrapping in foil may help to protect the parafilm from tears resulting from contact with aluminum foil.
**NOTE:** For small experiments, each plate can be wrapped in foil; for larger experiments it is recommended to wrap the entire storage box in foil for simplicity.
(7) Label the outside of plates with a piece of tape to hold the foil closed.
**CRITICAL:** From this point forward, plates should only be removed from foil in a dark room under green safe lights.

Place foiled plates vertically in the incubator at the desired temperature for germination and growth. In this case, 28°C was chosen as it is within the natural range for wheat growth and is similar to temperatures used in other studies (Wuest et al., 1999; Schramm et al., 2010). A box with vertical slots, as shown in **Figure 2**, can be used to hold plates in a vertical orientation. For large numbers of plates, it may be more efficient to enclose this box in aluminum foil rather than wrapping each plate individually.

### Transplanting Seedlings (Day 0)

**FIGURE 4:**
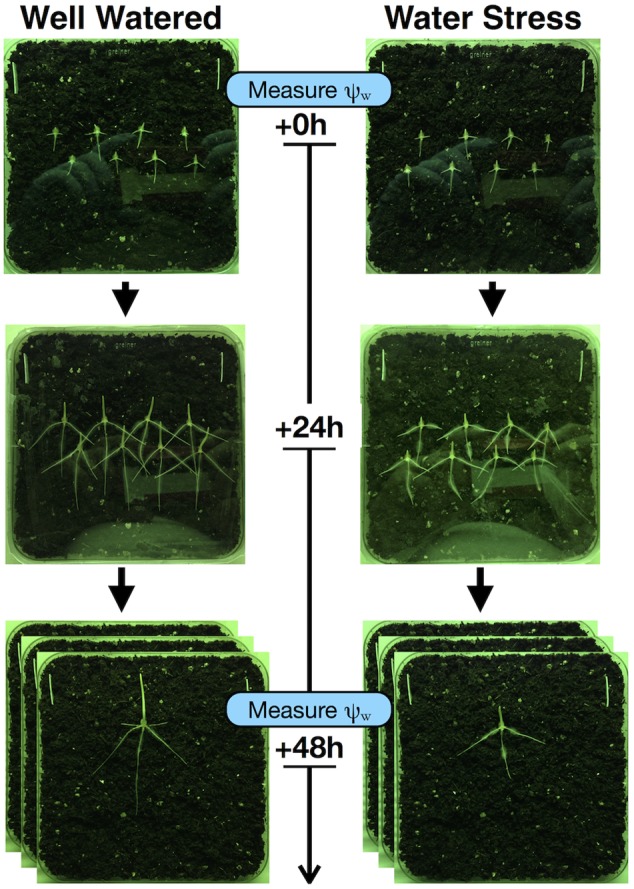
Data capture through digital photography at day 0, day 1, and day 2. Root measurements are captured by photography under green light at each time-point. Water potential is measured at the time of transplant and on the final day.

**NOTE:** Transplanting should occur in a dark room under green light in order to prevent exposure of roots to other wavelengths of light. Transplanting should occur as rapidly as possible: open source and destination plates, transfer and close.
**CRITICAL:** Seedlings selected for transfer should all be about the same size/root lengths (similar developmental stage) to allow fair comparison.

(1) Shortly before incubation has reached 30 h, prepare WW and WS soil plates. Prepare soil as before, but this time you will need three plates of WW soil and three plates of WS soil for transplanting.
**NOTE:** For preparation of WS plates, follow the same procedure as for WW plates but use the WS soil solution (PEG in 5 mM MES) with the desired concentration of PEG, instead of the WW soil solution. As PEG is not an idea solute, the water potential of a PEG-media cannot be calculated based on van’t Hoff’s law (Steuter, 1981). Precise water potentials must be determined for each experiment. An empty test plate should be prepared in advance of the experiment to determine the percentage of PEG to use for the desired water potential. Under our conditions, 5% PEG delivered approximately -0.1 MPa, of water-deficit stress, 10% PEG approximately -0.2 MPa, and 20% PEG approximately -0.65 MPa in soil-plates.
(2) At 30 h of incubation, transplant eight seedlings per plate into three plates per condition (WW or WS) as indicated in **Figure 4**. Seedlings should be in two rows of four and staggered near the center of the plate to allow space for both vertical and horizontal growth. The diagram in Supplementary Figure 3 is provided to serve as a to-scale guide for seedling placement.

### Digital Photography to Collect Root Length Data

Photograph plates on day 0 (directly after transplant), on day 1 (24 h after transplant), and on day 2 (48 h after transplant) (**Figure 4**). Digital photographs should be taken through the plastic lid under green light. Either use a stand to ensure the camera lens is parallel to the plate when photographing, or (if using a cell phone camera) select the square photograph option and turn the grid on, by making sure that the edges of the square frame and the edges of the square plate being photographed meet, you can ensure that the photograph is taken from parallel to the plate. This is important for accurate length measurements during image processing.

(1) Photograph at day 0 (0 h after transplant) in a dark room under green light. Use a digital camera or cell phone with green film over any lighted screen to prevent light contamination. Re-wrap plates with foil and replace vertically in incubator after photographing.
**NOTE:** At transplant all seedlings will have a shoot and three roots. These should all be clearly visible since the seedlings have just been transplanted.
(2) Photograph at day 1 (24 h after transplant) in a dark room under green light. Re-wrap with foil and replace vertically in incubator after photographing.
(3) Photograph at day 2 (48 h after transplant) in a dark room under green light. Root tissues can be harvested directly after photographing.
**NOTE:** Since day 2 is the final day, seedlings can be individually removed to a soil-plate without other seedlings one-at-a-time and photographed to capture the final root lengths. Any roots that were not fully visible on day 1 will, therefore, be clearly visible on day 2.
(4) Harvesting tissue at day 2 (48 h after transplant). Immediately after photography, the roots can be sampled for biochemical or molecular analyses by cutting the root at the desired distance from the tip and placing into liquid nitrogen.
**NOTE:** This process allows a pipeline of photography and sample collection on day 2 without allowing root tips to dry out. Both photography and tissue harvest must occur under green light in an otherwise dark room.
**CAUTION:** When using liquid nitrogen, ensure that the room used is well ventilated to prevent asphyxiation.

## Data Analysis

### Root Calling with SmartRoot in ImageJ

**FIGURE 5:**
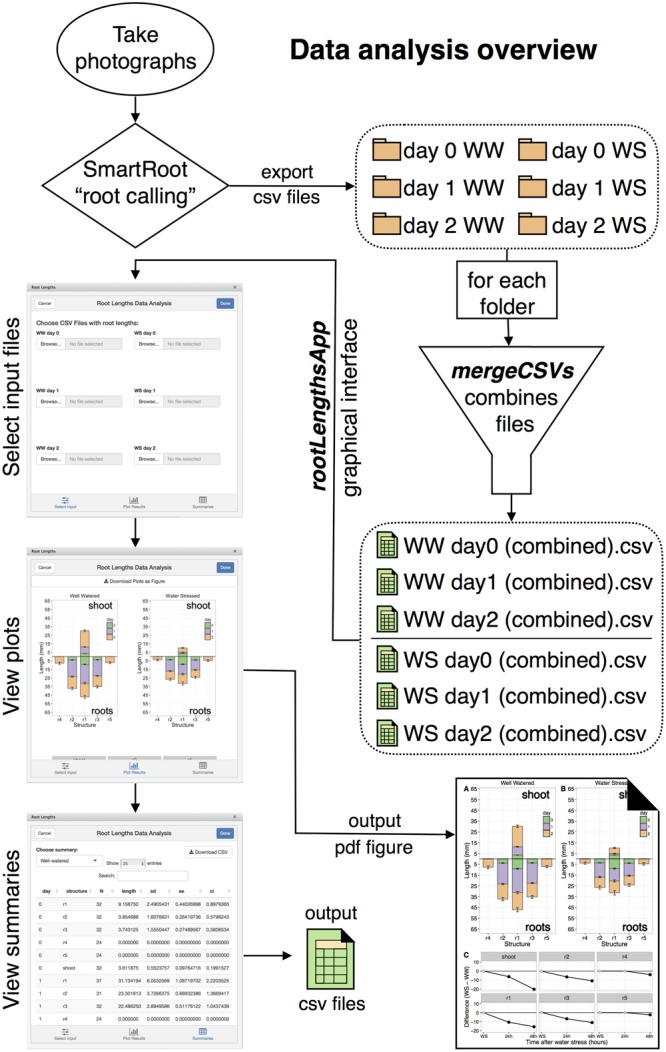
Diagram of the data analysis pipeline.

Data analysis starts with “root calling” using the SmartRoot plugin for Fiji or imageJ (**Figure 5**; Lobet et al., 2011; Schindelin et al., 2012; Schneider et al., 2012). Images imported into SmartRoot are converted to grayscale and inverted. The SmartRoot algorithm for root detection identifies potential roots and assists the user to trace the root by snapping nodes to areas where roots were detected. For unobstructed roots, root tracing can be automated from a single click for each root. For roots where auto-tracing fails or does not reach the tip of the root resulting from limited visibility or overlap, nodes can be added or adjusted manually to ensure accurate capture of root shape. Shoots can also be traced in a similar fashion to obtain shoot lengths. The SmartRoot software requires calibration to a line of known length to calculate millimeter lengths of the traced roots. This is achieved by tracing one of the reference 16 mm white plastic strips that was inserted into the soil at the start of the experiment. The strip is traced with the imageJ line tool and the length is set to 16 mm in the SmartRoot settings tab.

All roots and shoots must be labeled appropriately during root calling to facilitate automatic post-processing using the R package. There are a total of 24 seedlings for each condition, so each seedling should be numbered from 1 to 24 such that a root can be labeled “seed1rt1” or “seed1r1” to indicate root 1 of seedling 1 and a shoot can be labeled “seed24sht” or “seed24st” to indicate the shoot of seedling 24. All roots should be clearly visible in photographs from day 0, directly after transplant, and day 2, when each seedling is photographed individually. For photographs on day 1 the possibility exists for a root to be present, but not fully visible from the root growing around the edge of the plate or into the soil away from the transparent wall. In both of these cases, the root will not be counted toward *n*, the total number of replicates for a particular structure, and the mean will be calculated based on the remaining replicates. It is important to note that this will not affect the *n* of other structures. The most likely roots to be obscured would be root 2 or 4 of the left-most seedling and root 3 or 5 of the right-most seedling. For roots 1–3 and the shoot, the data processing algorithm has been designed to automatically remove these from calculations if data is not present for the root, so such roots should simply be skipped during root calling. For roots 4–6, since these were not present at day 0, it is necessary to differentiate a root 4 that is present but not fully visible (assigned NA), from one that is still absent on day 1 (assigned a value of zero). For root 4–6 only, roots with incomplete visibility should be traced and given a name such as “seed1r4_NA” to indicate a non-measurable root that will be excluded from data analysis. Roots that are absent will be automatically included in the calculation of mean with the value of 0 and will therefore also count toward *n*.

When root calling is complete, the table of root lengths for all traced roots and shoots in an image can be exported as a CSV file for data processing. Other measurements such as root angle, root area, or root diameter can also be exported in this manner if desired. The exported files must be named consistently using a naming scheme such as “20160413 Zak wt WW1 day0.csv” for the R function *mergeCVSs* to correctly merge the files and format the filename of the output file. The required features are the text “WW1” surrounded by spaces to indicate WW plate 1 (of three) and the file extension “.csv” at the end. “WS1” would indicate water-deficit stressed plate 1. There will be three image files for WW and WS for day 0 and day 1, but 24 image files for WW and WS for day 2, with each image file representing a single seedling, so for day 2 the naming format may include the seedling number s1–s24. The filename scheme is used only by the CSV merging function *mergeCSVs* to facilitate consistent output naming; however, the cultivar name placed after the first space in the filename will be used when the merged file is read into the *compileRootDF* function to populate the “genotype” column of the data frame that is produced.

### Data Processing and Plotting with MeasuRoots R Package

The measuRoots R package was written specifically for this pipeline so as to automate data analysis once root calling has been performed for each photograph and CSV files exported. This package is available online from github^2^. The measuRoots package is composed of six major R functions for data cleanup and analysis: *mergeCSVs, compileRootDF, rootPlot, summaryWSvsWW, plotDifferences*, and *rootLengthsApp*. The *mergeCSVs* function merges multiple CSV files from SmartRoot into a single CSV file to remove the need for manual copying and pasting to combine files. Root calling will produce six sets of CSV files that can be organized into six folders (**Figure 5**). WW and WS folders for day 0 and 1 will each contain three CSV files to be merged, while day 2 folders will each contain 24 files to be merged, one for each seedling. The first step after root calling is to merge all CSV files in each folder producing six CSV files containing combined data. The *mergeCSVs* function was written as an RStudio addin, so it can be run from the addins menu in the RStudio IDE^3^ or from the R console. The user is asked to select a file in the folder to be merged and merges all files in this folder into a single CSV with “(combined)” in the filename. These files can then be processed and analyzed using the remaining measuRoots functions.

**FIGURE 6:**
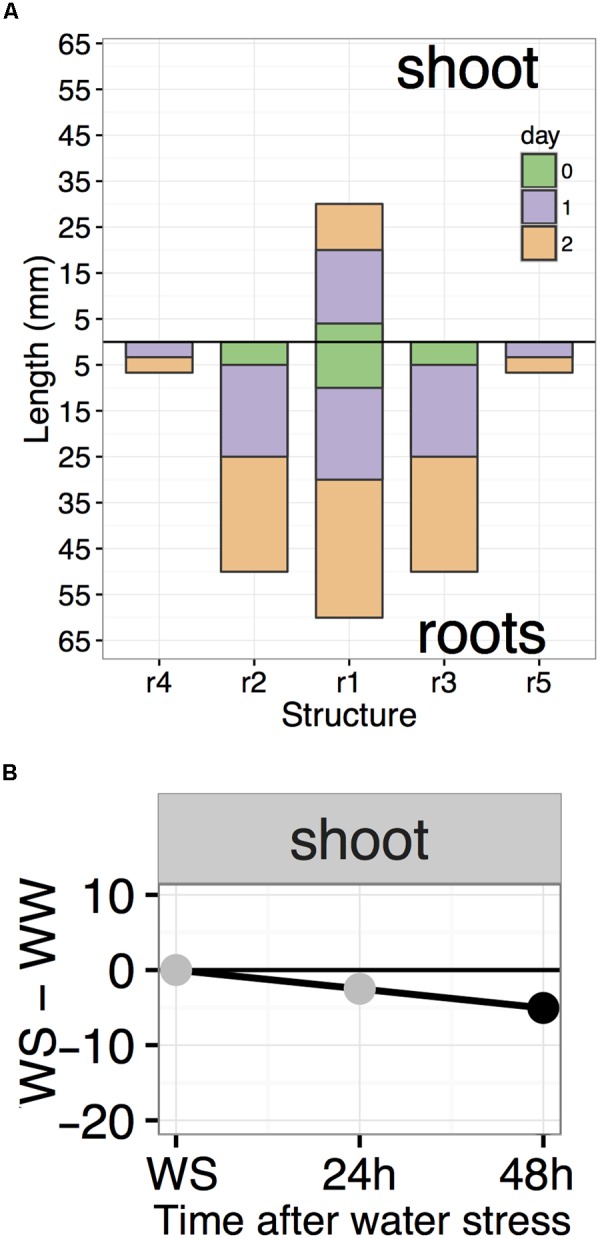
Examples of root plot and difference plot. **(A)** Roots in a root plots are organized in the same order as on the actual seedling. Root plots provide a computational representation of seedling shape by representing the lengths of each major structure. **(B)** A difference plot is broken into individual facets, each representing a single root or shoot. At each time-point, the difference in length between WS and WW is plotted with gray or black points. Black points indicate significance (*p* < 0.05).

Data analysis involves summarization of data into summary data frames which are then fed to specialized R functions that plot the results using the well-known ggplot2 plotting package (Wickham, 2009; R Core Team, 2016). The *compileRootDF* function processes day 0, 1, and 2 CSV files for a single condition and outputs a data frame which can be used by the *rootPlot* function for plotting “root plots” (**Figure 6A**). Root plots are bar plots with bars presented in the same organization as the roots and shoot of the seedling. Each downward-facing bar represents a root and the upward-facing bar represents the shoot. The changes in length from each time-point to the next are stacked to visually provide both the root length at each time-point and the amount of growth between two time-points. Root plots also provide a computational representation of the overall shape of a seedling. To produce a “difference plot” for all roots and the shoot, the *summaryWSvsWW* function is used to summarize the data and output a data frame for plotting with the *plotDifferences* function (**Figure 6B**). Difference plots present differences in root length between the WS and WW conditions at each time-point and also indicate the statistical significance of any difference observed. On the day of transplant, WS and WW seedlings should be at the same growth stage, with no significant difference in root or shoot lengths and points are plotted in gray. Under stress conditions, any significant differences between WS and WW are indicated by black points, based on a Mixed Model ANOVA using the afex R package with *post hoc* analysis using the lsmeans R package to determine *p*-values of comparisons at each timepoint with a default *p*-value cutoff of 0.05 (Lenth, 2016; Singmann et al., 2017). Plots output from *rootPlot* or *plotDifferences* functions are ggplot2 plots making it possible to save plots as variables and modify output characteristics (colors, line width, text, and so forth) using the Grammar of Graphics syntax used for other ggplot2 plots.

For ease-of-use, the measuRoots package includes the *rootLengthsApp* function, a graphical interface to automate all data analysis steps starting with the importing of the three WW and three WS CSV files. The *rootLengthsApp* function is designed as an addin to the RStudio IDE, thus it can be run by selection from the addins menu negating the need to enter R code. *rootLengthsApp* can be run either in a window within RStudio, as an RStudio gadget, or in a web browser locally. This interface provides point-and-click selection of input files for processing and displays plots and summary tables for each dataset (**Figure 5**). Figures can be exported in a single labeled vector pdf suitable for publication, while summary tables can be exported as CSV files that are viewable in most spreadsheet programs such as Microsoft Excel. *rootLengthsApp* can also return individual plots, which can be saved as R variables. The layout, formatting, or other plot options can then be modified using the same syntax as for any ggplot2 plot. In the case of seedlings with six, rather than five, total primary roots, a sixth root will be automatically added to plots. The data processing algorithm will ignore root six in plots unless more than three of the 24 seedlings have a sixth root present. For advanced users, this default cutoff and many other options with automatic default settings can be modified by providing the appropriate arguments to measuRoots functions when run from the R console.

## Anticipated Results

An experiment to compare the effects of water stress on root growth in seedlings of the Zak wheat cultivar was used to test this methodology and data analysis pipeline. Water stress was imposed to a ψ_w_ of -0.65 MPa with a 20% PEG solution and compared to seedlings under WW conditions. Soil ψ_w_ of the WS treatment was consistent from the time of transplant (-0.65 ± 0.01) to 48 h after transplant (-0.65 ± 0.05), based on four measurements taken at each timepoint. Although the Decagon WP4C dewpoint hygrometer is less accurate as ψ_w_ approaches 0, three measurements were also taken for WW at day 0 (-0.02 ± 0.01) and day 2 (0.00 ± 0.02) to confirm WW conditions.

**FIGURE 7:**
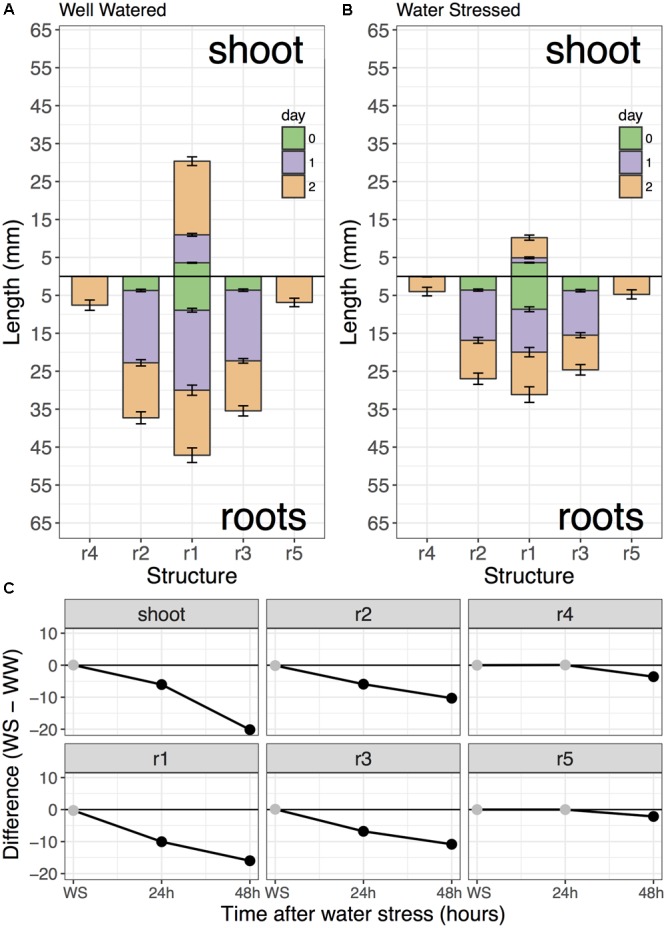
Comparison of WW and WS seedling root growth. This figure was output directly from the *rootLengthsApp* function. Well-watered **(A)** Zak wheat was compared to –0.65 MPa water stressed **(B)** seedlings. **(C)** Difference plot shows the difference in root or shoot length at each time-point and indicates significance with black dots (*p* < 0.05).

**Figure 7** shows the results of the root length data analysis for this comparison and is an example of a pdf figure exported through the graphical interface of the *rootLengthsApp* function. The overall effects of the water stress treatment can be clearly observed from the reduced growth of the WS seedling (**Figures 7A,B**). Water stress had a greater inhibitory effect on shoot growth than root growth, although both grew less under WS conditions. Interestingly, the rate of root growth for roots 1–3 appears greatest at a time between transplant and 24 h after transplant for both WW and WS seedlings, while roots 4 and 5 do not emerge until later than 24 h after transplant, which may be a cultivar specific trait. For increased resolution of changes in root growth rate, future measurements could be taken at 12 h intervals, particularly to capture root length at the time-point 12 h after transplant.

Using difference plots, it was possible to confirm that seedlings for both WW and WS conditions were at approximately the same starting growth stage, since no significant differences in structure length for roots or shoot were observed (**Figure 7C**). Due to the moderate water stress imposed by -0.65 MPa soil ψ_w_, significant growth differences were apparent by 24 h after transplant in root 1 through 3 and the shoot. These differences were more striking by 48 h and associated with a higher degree of significance, as shown in **Table 1**.

**Table 1:** Summary table of data for difference plot comparing WS (-0.65 MPa) and WW root growth.

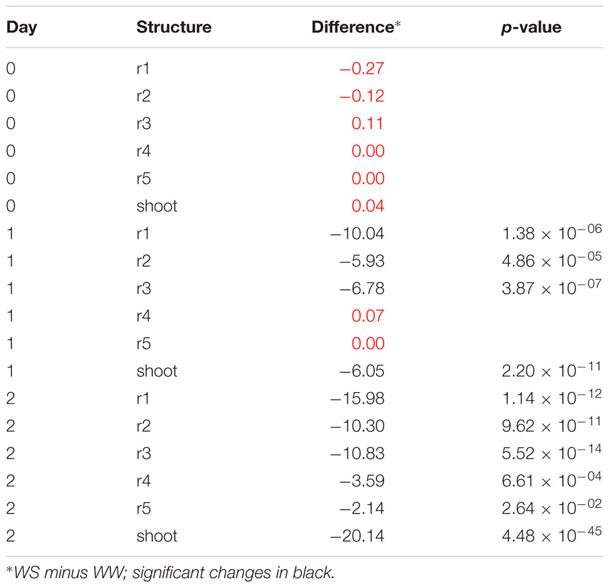

## Discussion

The soil-plate method presents an efficient pipeline for root growth measurement under water stress allowing an experiment to be set up in an hour and measurements collected quickly at each timepoint via photography. This method allows controlled experiments to be conducted on soil without compromising factors such as exposure of roots to white light. Experiments utilizing the soil-plate method do not require specialized robotics or growth chamber facilities aside from a laboratory incubator and can be easily scaled up. Apart from the WP4C dewpoint hygrometer, no special equipment is required making this setup accessible to most research labs. Data collection via digital photography and SmartRoot image processing resulted in the ability to capture root lengths at a static point in time and provided more accurate fine distance measurements, particularly for roots that did not grow with perfect linearity (a common occurrence). The open source data analysis pipeline means that results can be viewed immediately once root calling has been performed. The point-and-click graphical interface makes this pipeline accessible to anyone without programming skills.

There are many available methods and associated software packages for conducting root growth experiments and each has their own advantages and disadvantages. One advantage of this system is that all software used here is free. Root calling is semi-automated, while some software (e.g., WinRhizo) has fully automated root calling. However, the tradeoff of a less automated approach is that accuracy is very high and the chance of missing roots or parts of roots is very low. Throughput is also lower than a fully automated method, but is higher than methods that involve hand-tracing roots on rhizobox walls or tracing paper. Furthermore, this method benefits over methods that require scanning since scanners expose roots to high intensity white light during scanning, and over methods than use excavation of roots (shovelomics), as time course measurements are possible. This method does not, however, test true field conditions as shovelomics methods do, nor does it allow manipulation of water deficit gradually over time as hydroponics methods allow. Thus, the protocol presented here describes a method that is somewhere between lab and field which allows measurements under controlled conditions, but still maintains the use of non-living soil.

While this study outlines the application of the soil-plate protocol for assaying water-deficit stress, it can be adapted easily for assaying response to other root treatments, such as hormones or hormone inhibitors, which are generally difficult to assay under water deficit in soil since application generally occurs as a liquid. This opens up a range of possibilities for future experiments that combine water stress and hormone or inhibitor treatments. Additionally, we have confirmed that maize and soybean can be grown in a similar manner, suggesting that the use of this method is not limited to specific crops or experimental plant models (Supplementary Figure 4). For older seedlings or for faster growing plants such as maize, it may be valuable to utilize 24 cm square plates (Thermo Fischer item # 240845) instead of the 12 cm plates used here. This methodology can also be used to investigate seedling root architecture traits like root angle or finer traits like root hair characteristics.

## Author Contributions

MO provided the initial research design and obtained funding. SN developed methodology and computational tools and performed all experiments and analyses. Both authors contributed to the final methodology design, and to the writing of this article.

## Conflict of Interest Statement

The authors declare that the research was conducted in the absence of any commercial or financial relationships that could be construed as a potential conflict of interest.

## Footnotes

1 https://github.com/publiclab/spectral-workbench

2 https://github.com/bakuhatsu/measuRoots

3 www.rstudio.com
