# Supplementary material for: A Soil-Plate Based Pipeline for Assessing Cereal Root Growth in Response to Polyethylene Glycol (PEG)-Induced Water Deficit Stress
Source: Front Plant Sci. 2017 Jul 19;8:1272. doi: 10.3389/fpls.2017.01272 (PMC5515875; doi:10.3389/fpls.2017.01272)
Supplement: Supplementary file 1 [file Supplementary_Material.PDF]

frontiers

Supplementary Material

A Soil-plate Based Pipeline for Assessing Cereal Root Growth in Response to Water Stress

Sven K. Nelson*, Melvin J. Oliver

***Correspondence:** Sven K. Nelson: Sven.Nelson@ars.usda.gov

1. Supplementary Figures

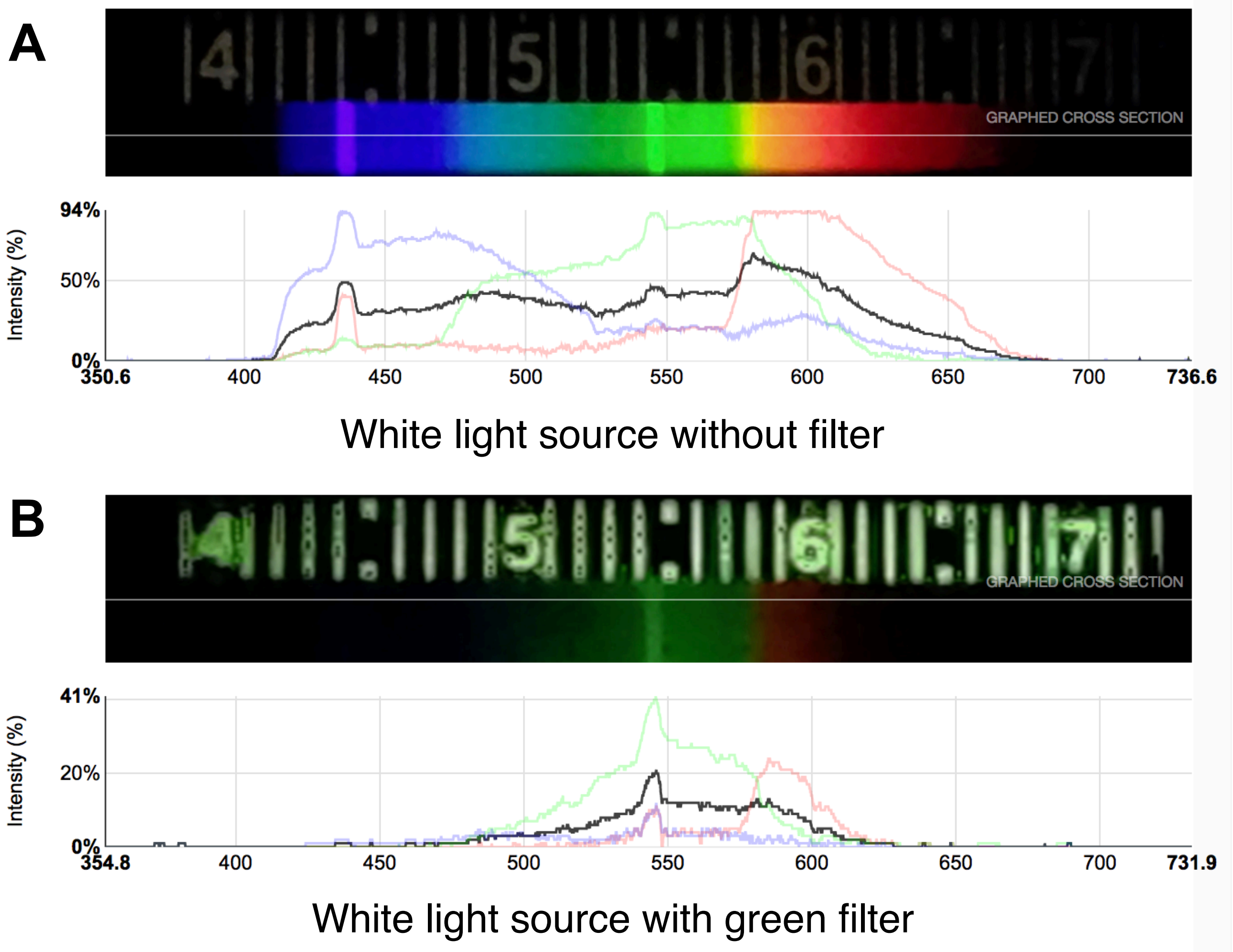

Supplementary Figure 1. Wavelengths of the unfiltered white light source **(A)** and of the light source filtered for green light using a green transparent film **(B)**.

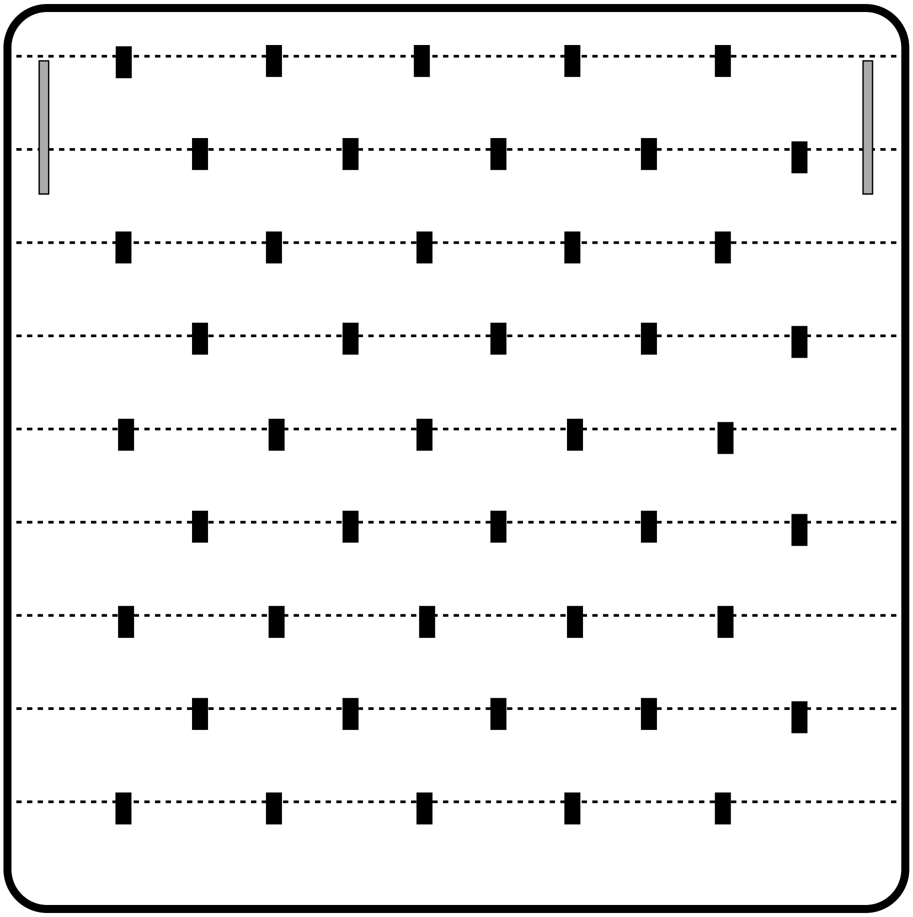

Supplementary Figure 2. Actual size printable diagram to aid in seed placement during initial plating.

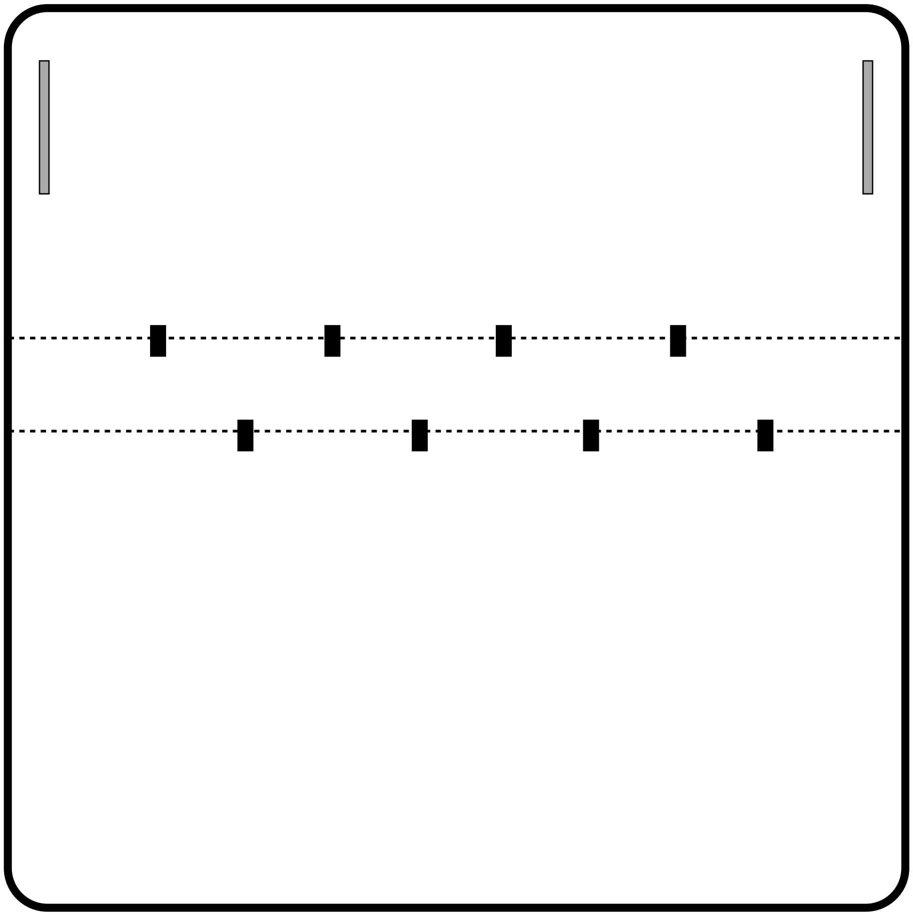

Supplementary Figure 3. Actual size printable diagram to aid in seedling placement during transplant step.

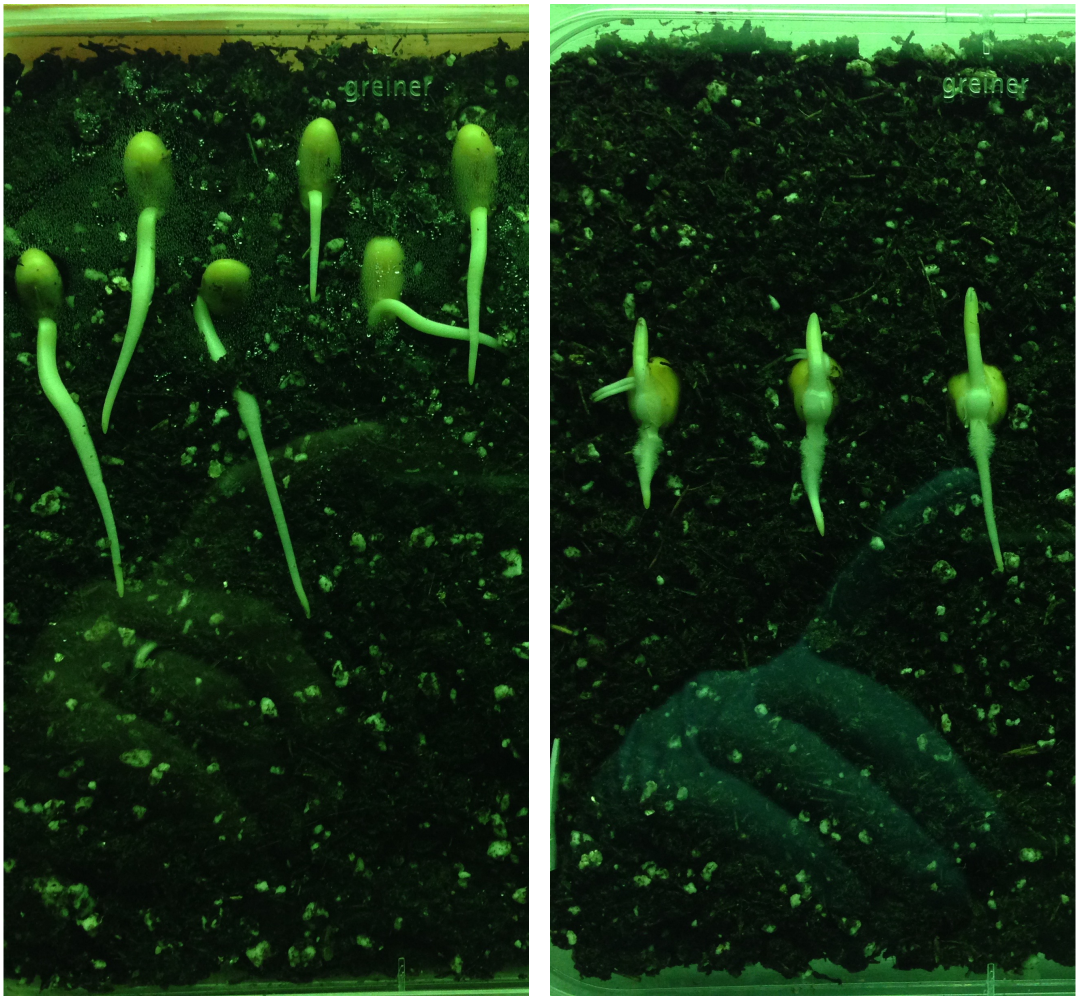

Soybean **Maize**

Supplementary Figure 4. Tests of soil-plates with soybean and maize.
